# Genome-wide detection of CNVs and their association with performance traits in broilers

**DOI:** 10.1186/s12864-021-07676-1

**Published:** 2021-05-17

**Authors:** Anna Carolina Fernandes, Vinicius Henrique da Silva, Carolina Purcell Goes, Gabriel Costa Monteiro Moreira, Thaís Fernanda Godoy, Adriana Mércia Guaratini Ibelli, Jane de Oliveira Peixoto, Maurício Egídio Cantão, Mônica Corrêa Ledur, Fernanda Marcondes de Rezende, Luiz Lehmann Coutinho

**Affiliations:** 1 Department of Animal Science, University of São Paulo (USP), Luiz de Queiroz College of Agriculture (ESALQ), Piracicaba, São Paulo 13418-900 Brazil; 2 Unit of Animal Genomics, GIGA-R, University of Liège, Liège, Belgium; 3 Embrapa Suínos e Aves: Empresa Brasileira de Pesquisa Agropecuária Suínos e Aves, Concórdia, Santa Catarina Brazil; 4 Animal Sciences Department, University of Florida, Gainesville, FL 32608 USA

**Keywords:** GWAS, Performance, CNVs, QTLs, qPCR

## Abstract

**Background:**

Copy number variations (CNVs) are a major type of structural genomic variants that underlie genetic architecture and phenotypic variation of complex traits, not only in humans, but also in livestock animals. We identified CNVs along the chicken genome and analyzed their association with performance traits. Genome-wide CNVs were inferred from Affymetrix® high density SNP-chip data for a broiler population. CNVs were concatenated into segments and association analyses were performed with linear mixed models considering a genomic relationship matrix, for birth weight, body weight at 21, 35, 41 and 42 days, feed intake from 35 to 41 days, feed conversion ratio from 35 to 41 days and, body weight gain from 35 to 41 days of age.

**Results:**

We identified 23,214 autosomal CNVs, merged into 5042 distinct CNV regions (CNVRs), covering 12.84% of the chicken autosomal genome. One significant CNV segment was associated with BWG on GGA3 (*q*-value = 0.00443); one significant CNV segment was associated with BW35 (*q*-value = 0.00571), BW41 (*q*-value = 0.00180) and BW42 (*q*-value = 0.00130) on GGA3, and one significant CNV segment was associated with BW on GGA5 (*q*-value = 0.00432). All significant CNV segments were verified by qPCR, and a validation rate of 92.59% was observed. These CNV segments are located nearby genes, such as *KCNJ11, MyoD1* and *SOX6*, known to underlie growth and development. Moreover, gene-set analyses revealed terms linked with muscle physiology, cellular processes regulation and potassium channels.

**Conclusions:**

Overall, this CNV-based GWAS study unravels potential candidate genes that may regulate performance traits in chickens. Our findings provide a foundation for future functional studies on the role of specific genes in regulating performance in chickens.

**Supplementary Information:**

The online version contains supplementary material available at 10.1186/s12864-021-07676-1.

## Background

*Gallus gallus* is an excellent biological model organism for genetic studies [1] and a species of considerable economic relevance worldwide. In 2019, global poultry meat consumption was estimated at 97,000 tons [2], being one of the main sources of protein for humans. Understanding the genetic architecture of performance-related traits may contribute to the development of new genomic strategies to increase production efficiency and sustainability of the chicken industry.

Significant advances have been achieved on chicken genetics [3] since the landmark publication of the first reference genome [4], which has been continuously updated with the most recent genome assembly (GRCg6a) released in 2018. Variations in the genome, especially single nucleotide polymorphisms (SNPs), are known to be associated with phenotypic variation [5]. However, structural variations, such as copy number variations (CNVs) have been increasingly studied and associated with quantitative traits of economic interest in livestock [6–9].

CNVs associated with phenotypes of economic interest are promising targets for animal breeding programs [10]. They are defined as large DNA fragments (conventionally > 1 kb) that, due to deletion or duplication events, display variable copy number between individuals of a population [11]. When compared to SNPs, CNVs encompass more total bases and seem to have a higher mutation rate and potentially greater effects on gene structure, gene regulation and consequently gene expression [12].

Various techniques are available for CNVs detection in humans and other animal species [13]. Most of them depend on the analysis of signal intensity along the genome, such as the comparative genomic hybridization array (aCGH) [14] and high-density SNP chips [15]. Although sequencing-based CNV analyses pipelines have been developed and seem to be a viable alternative [16], SNP chips have been commonly used for CNV detection [8, 17]. This technology allows CNVs identification due to the abnormal hybridization that occurs for SNPs located in CNV regions (CNVRs) [15]. Simultaneous measurement of both signal intensity variations, measured for each allele of a given SNP, and changes in allelic composition (i.e. B allele frequency) allow the detection of both copy number changes and copy-neutral loss-of-heterozygosity (LOH) events [15].

Several factors, such as detection algorithm, genotyping platform, SNP density and population genetic background may impact CNV scanning performance [18]. Indeed, different algorithms used for CNV detection may demonstrate variable sensitivity, consistency and reproducibility, especially for commercial SNP arrays [19], such as Illumina and Affymetrix SNP chips. One of the most prominent algorithms for CNV detection is the PennCNV software [20], which has been widely applied in several studies on livestock species, including chickens [7], horses [21], pigs [22], cattle [6] and sheep [17]. Moreover, PennCNV has better consistency when compared to other CNV calling algorithms [19]. Nevertheless, CNVs identified through SNP-chip platforms can be associated with a considerable rate of false negative and positive results [18]. Therefore, the quantitative polymerase chain reaction (qPCR) is commonly used for CNV validation, being a molecular method to confirm computationally identified loci [8, 23].

In chickens, several studies have identified quantitative trait loci (QTL) and positional candidate genes flagged by SNPs significantly associated with traits of economic interest such as performance, carcass and abdominal fat [24, 25]. Unsurprisingly, the number of CNV-focused studies is increasing in chicken populations as well [7, 26]. CNVs associated with late feathering [27], pea-comb phenotype [28], dermal hyperpigmentation [29], dark brown plumage color [30] and resistance/susceptibility to Marek’s disease [31] have been reported. None CNV-association study for performance traits in chickens has been described yet.

Herein, we identified CNVs in the genome of a broiler population, performed a CNV-based GWAS for performance traits and validated associated CNV segments by qPCR. In addition, we identified performance-related genes overlapping significant CNV segments to establish relationships between structural genomic variation and such phenotypes.

## Results

### CNV identification

After applying the initial quality control filters, 223 individuals out of 1461 genotyped chickens from the TT Reference Population presented DishQC< 0.82 and call rate < 97%, and were excluded from further analyses. Therefore, individual-based CNV calls were performed on the remaining 1238 samples. Pedigree information on father-mother-offspring trio was used to update the CNV status for the trios, generating more accurate CNV calls [20]. From the total of 1238 chickens, 709 trios were determined based on complete family information available. Then, the trio-based CNV calling using 779 animals, represented by 709 trios, consisting of 14 sires, 56 dams and 709 offspring, was performed. Several families with incomplete information could not be used as PennCNV is not able to handle trios with missing sire or dam genotypes. After quality control filtering and removal of duplicated CNVs from the dataset, we identified 23,214 unique autosomal CNVs, including 2905 deletions and 20,309 duplications. Finally, a total of 614 chickens had at least one CNV call after the quality control process.

### CNVR compilation

CNVRs represent the concatenation of overlapping CNVs into a consensus genomic region. CNVs showing overlap of at least one base pair among samples in this population were summarized across all individuals into CNVRs. After filtering, 23,214 individual CNVs were merged into 5042 distinct CNV regions, which cover 12.84% (136.75 million of base pairs - Mb) of the chicken autosomal genome. The number of regions with copy loss and gain were 424 and 4105, respectively. The presence of both types was observed in 513 regions. The CNVRs had variable sizes ranging from 0.14 kb to 760 kb with an average size of 27.12 kb. The number of chickens with CNVs mapped onto a given CNVR ranged from 1 (0.13%) to 348 (44.67%) from the total of 614 chickens. We identified 656 CNVRs occurring in more than 1% of the population (i.e. ‘polymorphic CNVRs’, as suggested by Itsara et al. [32]). The relative chromosome coverage by CNVRs ranged from 1.55% for GGA24 to 18.38% for GGA2, while the absolute genomic length overlapped by CNVRs varied from 0.10 Mb for GGA24 to 35.98 Mb for GGA1. Detailed information of all CNVRs detected in our population is provided in Additional file 1.

### Association of CNV segments with performance traits

**Fig. 1:**
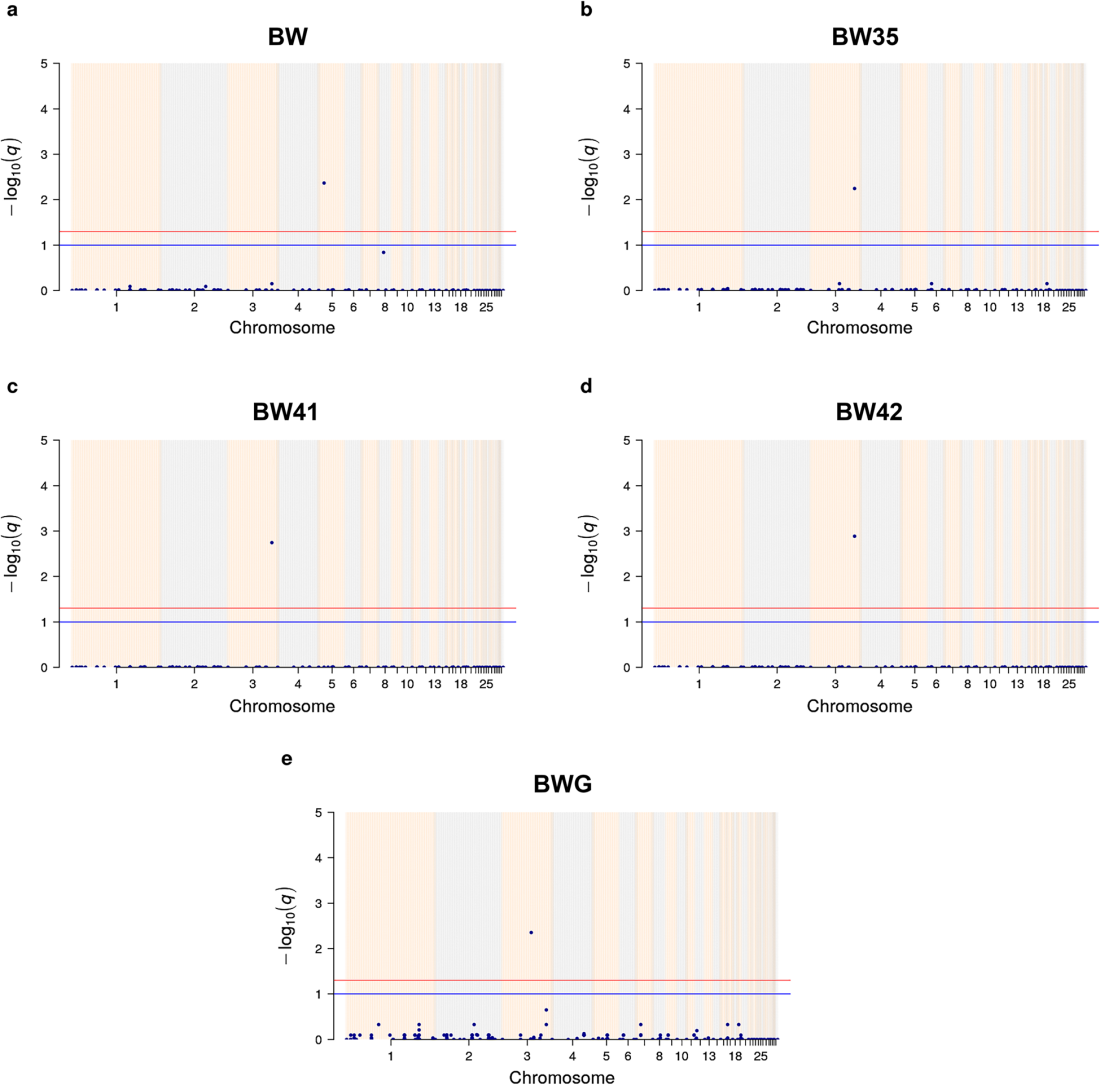
Manhattan plots for CNV segments across the 33 autosomal chromosomes associated with **a** birth weight, **b** body weight at 35 days, **c** body weight at 41 days and **d** body weight at 42 days and **e** body weight gain. The X-axis represents the somatic chromosomes, and Y-axis shows the corresponding -log_10_ *q*-value. Red and blue lines indicate FDR-corrected *p-*values of 0.05 and 0.1, respectively

Genome-wide association studies were performed to investigate significant associations of CNV segments, named as CNV-based GWAS, with eight performance-related traits measured in our population: BW, BW21, BW35, BW41, BW42, FI, FCR and BWG. Manhattan plots for CNV segments across the 33 autosomal chromosomes associated with performance traits are presented in Fig. 1. Note that the FDR method for multiple testing correction shrunk the -log_10_(*q*-value) for non-significant CNV segments towards zero, while it magnified the -log_10_(*q*-value) for significant associated CNV segments. The Manhattan plots of the raw *p*-values for CNV segments across the 33 autosomal chromosomes associated with performance traits and the QQplots for BW, BW35, BW41, BW42 and BWG are in Additional files 2 and 3, respectively.

There were three distinct CNV segments classified as losses and significantly associated (*q*-value< 0.05) with BWG, BW35, BW41, BW42 and BW (Table 1). One CNV segment was significantly associated with BWG (*q*-value = 0.00443); one CNV segment was significantly associated with BW35 (*q*-value = 0.00571), BW41 (*q*-value = 0.00180) and BW42 (*q*-value = 0.00130), and one CNV segment was significantly associated with BW (*q*-value = 0.00432). It is interesting to highlight that the significant CNV segment associated with BW35, BW41 and BW42 was the same (GGA3:97801202–97809208). Note that none significant CNV segments associated with BW21, FI and FCR were detected.

**Table 1:** Characterization of significant CNV segments associated with performance traits in the TT Reference Population

Trait^a^	GGA: first–last position^b^	Number of genes/window^c^
BWG	3: 64169030–64171297	16
BW35	3: 97801202–97809208	3
BW41	3: 97801202–97809208	3
BW42	3: 97801202–97809208	3
BW	5: 12059966–12062666	13

^a^BWG: body weight gain from 35 to 41 days; BW35: body weight at 35 days; BW41: body weight at 41 days; BW42: body weight at 42 days; BW: birth weight

^b^Map position based on GRCg6a chicken genome assembly

^c^Number of annotated genes within a 1-Mb window of each significant CNV segment associated with performance traits in the TT Reference Population, based on Ensembl Genes 101 Database (https://www.ensembl.org/biomart/martview/)

**Fig. 2:**
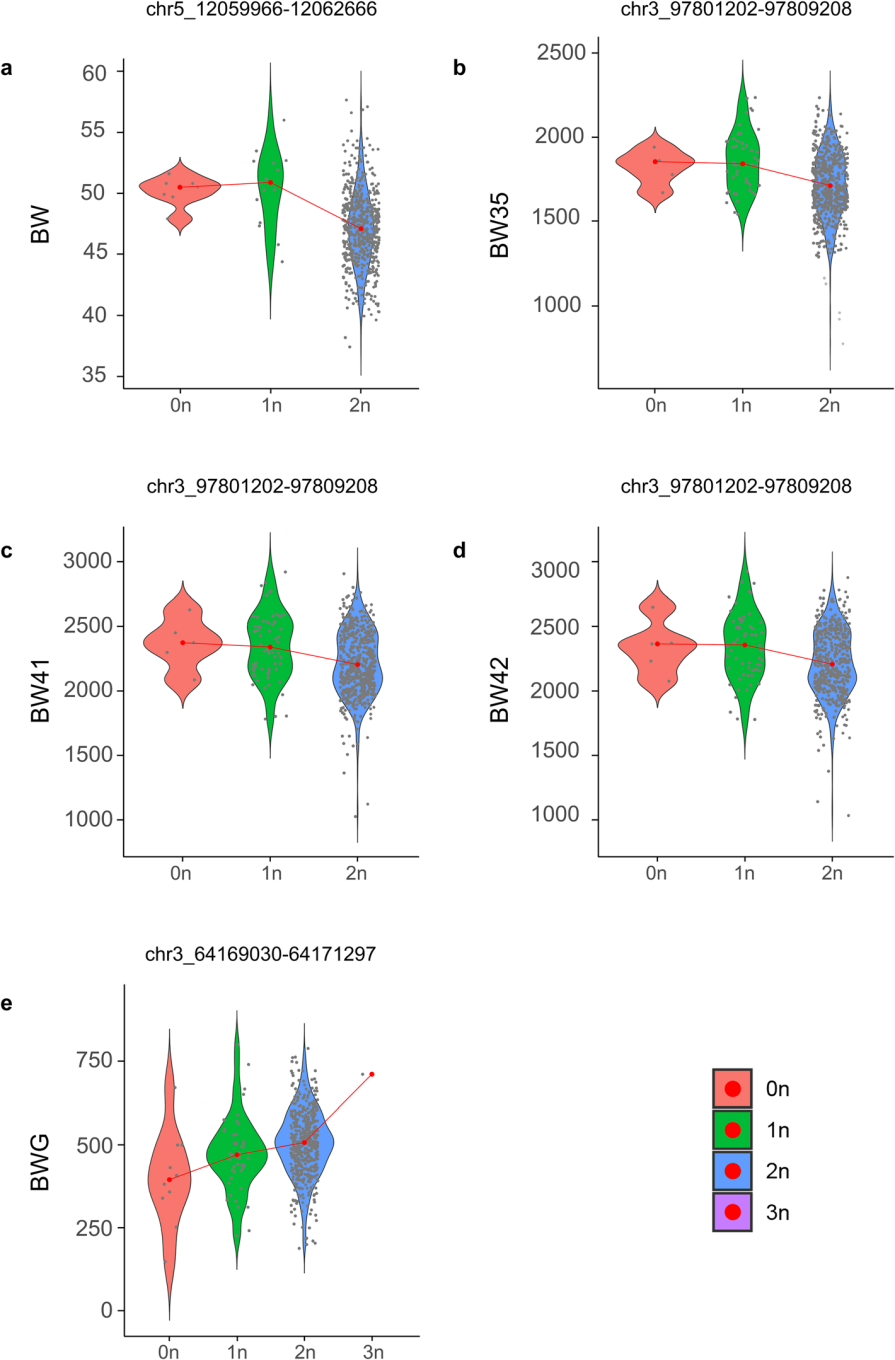
**a** Birth weight, **b** body weight at 35 days, **c** body weight at 41 days and **d** body weight at 42 days and **e** body weight gain distribution in each CN state for the significant CNV segment. Each dot represents an animal in the corresponding copy number state (0-3n) on the X-axis and the observed phenotypic value on the Y-axis. The legend on the right displays the color code for the CN state. See the main text for a detailed description of each segment

In Fig. 2, each dot represents an animal in the corresponding copy number state (0-3n) on the X-axis and the observed phenotypic value on the Y-axis. For the significant CNV segment associated with BW (GGA5: 12059966–12062666), a decrease in copy number is associated with heavier birth weight. The same trend was observed for the significant CNV segment associated with BW35, BW41 and BW42 (GGA3: 97801202–97809208), i.e. higher copy number was observed in animals with lower body weight. Conversely, the significant CNV segment associated with BWG (GGA3: 64169030–64171297) displayed an opposite behavior.

### qPCR validation

**Fig. 3:**
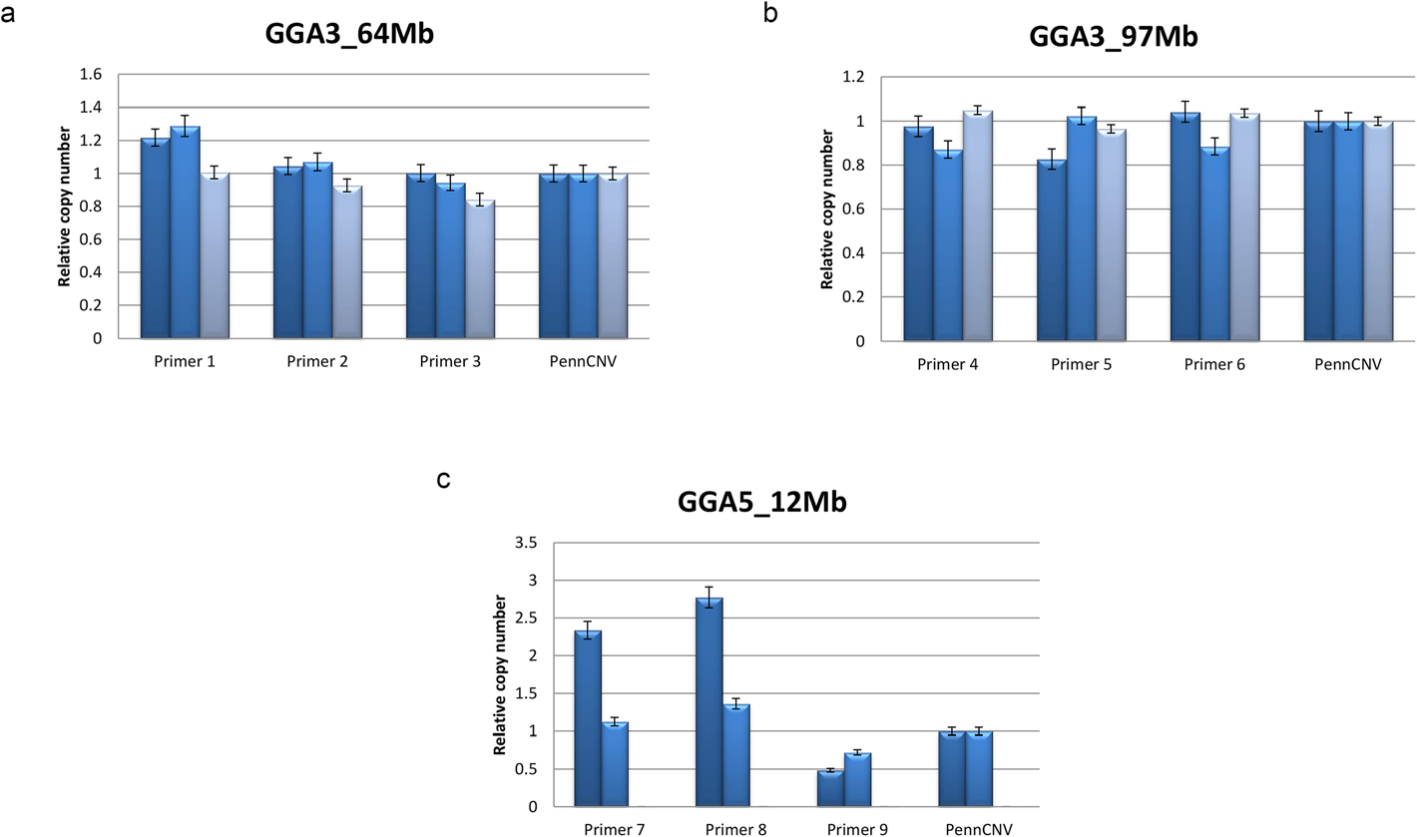
Quantitative PCR was carried out for significantly associated CNV segments on **a** GGA3 at 64 Mb, **b** GGA3 at 97 Mb and **c** GGA5 at 12 Mb using two groups (control (2n) and experimental) with three different animal samples per group and three distinct primer pairs per CNV. In each panel, bars in different colors represent distinct experimental animals for each segment. The right-most bars depict the relative copy number estimated for each animal in PennCNV. Each bar was calculated from three technical replicates. The error bars show the minimum and maximum value encountered among the replicates

Since that CNV breakpoints depend on the segmentation algorithm used, some variation on CNV segment detection between PennCNV and qPCR is expected. The qPCR results (Fig. 3) revealed a validation rate of 92.59%, which confirms the existence of CNV segments that have been associated with performance traits. In addition, it revealed that the CNV type was concordant between both methods for most of the samples, except for the first sample with primers 7 and 8. Note that for CNV segments where at least one breakpoint was within the target segment, PennCNV results were confirmed by qPCR. It is important to mention that the third tested animal had a copy number status estimated by PennCNV of 0n for the CNV segment on GGA5. Primer information and validation rates are presented in Additional files 4 and 5, respectively.

### CNV segments overlapping known QTLs

The significant CNV segments associated with body weight gain (GGA3: 64169030–64171297) overlapped with previously mapped QTLs for body weight at 49 days of age (QTL #30854, [33]), comb weight (QTL #127114, [34]), residual feed intake (QTL #64556, [35]), and testis weight (QTL #213559, [36]). The significant CNV segment associated with BW35, BW41 and BW42 (GGA3: 97801202–97809208) also overlapped with two QTLs described above (QTL #30854 and #127114). Moreover, both significant CNV segments overlapped with 18 out of 27 previously published QTLs for growth-related traits mapped in the Embrapa F2 Chicken Resource Population ([37], Table 2). None previously reported QTLs overlapped with the CNV segment significantly associated with birth weight (GGA5: 12059966–12062666).

**Table 2:** CNV segments associated with performance traits overlapping QTL regions previously mapped for growth-related traits

CNV segments (GGA: first-last position^a^)	QTL_IDs	Associated trait
3: 64169030–64171297; 3: 97801202–97809208	**QTL #1979**	**Body_weight**
3: 64169030–64171297; 3: 97801202–97809208	**QTL #1980**	**Body_weight**
	QTL #7184	Body_weight_(41_days)
3: 64169030–64171297; 3: 97801202–97809208	**QTL #7180**	**Body_weight_(35_days)**
3: 64169030–64171297; 3: 97801202–97809208	**QTL #55904**	**Body_weight_(35_days)**
3: 64169030–64171297; 3: 97801202–97809208	**QTL #55929**	**Growth_(0-35_days)**
	QTL #24377	Body_weight_(35_days)
	QTL #24378	Body_weight_(41_days)
	QTL #24379	Body_weight_(42_days)
	QTL #7167	Body_weight_(1_day)
	QTL #7171	Body_weight_(35_days)
	QTL #7174	Body_weight_(41_days)
3: 64169030–64171297	**QTL #1957**	**Body_weight**
	QTL #7156	Body_weight_(35_days)
	QTL #7161	Body_weight_(41_days)
3: 64169030–64171297; 3: 97801202–97809208	**QTL #1961**	**Body_weight**
3: 64169030–64171297; 3: 97801202–97809208	**QTL #1962**	**Body_weight**
3: 64169030–64171297	**QTL #6611**	**Body_weight_(112_days)**
3: 64169030–64171297	**QTL #6612**	**Body_weight_(200_days)**
3: 64169030–64171297	**QTL #6610**	**Body_weight_(8_days)**
3: 64169030–64171297	**QTL #6613**	**Growth_(1-8_days)**
3: 64169030–64171297; 3: 97801202–97809208	**QTL #9420**	**Body_weight_(63_days)**
3: 64169030–64171297	**QTL #11768**	**Body_weight_(49_days)**
3: 64169030–64171297	**QTL #11772**	**Body_weight_(63_days)**
3: 64169030–64171297	**QTL #1969**	**Body_weight**
3: 64169030–64171297	**QTL #1972**	**Body_weight**
3: 64169030–64171297	**QTL #9127**	**Growth_(post-challenge)**

CNV segments significantly associated with performance traits located within QTL regions for growth-related traits [37]. QTLs that overlap genomic intervals covered by CNV segments associated with body weight gain (GGA3: 64169030–64171297) and/or body weight at 35, 41 and 42 days (GGA3: 97801202–97809208) are highlighted in bold text

^a^Map position based on GRCg6a chicken genome assembly (NCBI)

### Identification of regulatory elements

We investigated the presence of CpG islands within the significant CNV segments. However, no CpGs were identified on such regions (Additional file 6). Moreover, we found that the significant CNV segment on chromosome 5 (GGA5:12059966–12062666), previously associated with birth weight, is located nearby the *KCNJ11* gene, approximately 3.4 kb downstream of the gene start site. Our analysis of ChIP-seq data for H3K27ac of chicken skeletal muscle, an indicator of cis-regulatory elements, like active enhancers [38], showed overlapping of a H3K27ac enriched region with the aforementioned CNV segment (Additional file 6).

### Candidate genes and gene-set analysis

A total of 32 genes, including *KCNJ11*, *MyoD1* and *SOX6*, were annotated within a 1-Mb window in genomic regions defined by significant CNV segments associated with BWG, BW35, BW41, BW42 and BW (Table 3). A list with detailed information about the 32 genes is provided in the Additional file 7.

**Table 3:** List of notated genes within a 1-Mb window of significantly associated CNV segments

GGA: first-last position^a^	Associated trait (s)^b^	Gene Name (Aliases)	Ensembl Gene ID^c^	Entrezgene ID^d^
3: 64169030–64171297	BWG	RFX6	ENSGALG00000014918	421737
		GPRC6A	ENSGALG00000014925	428620
		FAM162B	ENSGALG00000019941	100857953
		KPNA5	ENSGALG00000014937	421738
		ZUFSP (ZUP1)	ENSGALG00000014940	421739
		SOT3A1L	ENSGALG00000014950	421740
		RWDD1	ENSGALG00000014953	421741
		FAM26D (CALHM4)	ENSGALG00000014955	421742
		TRAPPC3L	ENSGALG00000028539	421743
		FAM26E (CALHM5)	ENSGALG00000038162	769904
		FAM26F (CALHM6)	ENSGALG00000014962	421744
		DSE	ENSGALG00000014963	421745
		NT5DC1	ENSGALG00000014964	421746
		COL10A1	ENSGALG00000014965	100858979
		FRK	ENSGALG00000014979	421747
		HS3ST5	ENSGALG00000026594	428621
3: 97801202–97809208	BW35, BW41, BW42	GREB1	ENSGALG00000016455	421944
		LPIN1	ENSGALG00000016456	421945
		TRIB2	ENSGALG00000016457	378919
5: 12059966–12062666	BW	SOX6	ENSGALG00000006074	423068
		C5H11orf58	ENSGALG00000006077	395520
		PLEKHA7	ENSGALG00000029679	423069
		RPS13	ENSGALG00000006096	414782
		PIK3C2A	ENSGALG00000006121	423070
		NUCB2	ENSGALG00000006147	423071
		KCNJ11	ENSGALG00000020505	428846
		ABCC8	ENSGALG00000006172	423072
		USH1C	ENSGALG00000006192	423073
		FTL	ENSGALG00000028696	378899
		MYOD1	ENSGALG00000006216	374048
		KCNC1	ENSGALG00000006220	423076
		SERGEF	ENSGALG00000006231	423077

^a^Map position based on GRCg6a chicken genome assembly (NCBI)

^b^BWG: body weight gain from 35 to 41 days, BW35: body weight at 35 days, BW41: body weight at 41 days, BW42: body weight at 42 days, BW: birth weight

^c^Ensembl gene ID based on GRCg6a genome assembly (Ensembl Genes 101 Database)

^d^NCBI gene ID based on GRCg6a genome assembly (http://www.ncbi.nlm.nih.gov/gene)

Gene enrichment analysis was performed using WebGestalt to search for biological processes, cellular components and molecular functions. WebGestalt top 10 most relevant enriched categories for Biological Process, Cellular Component and Molecular Function, based upon genes annotated to each category, can be observed in Table 4. Noticeably, the most relevant enriched categories for biological processes, such as regulation of striated muscle cell differentiation, regulation of muscle cell differentiation and regulation of muscle tissue development, are directly implicated in muscle growth and development.

**Table 4:** WebGestalt to 10 most relevant enriched categories for Biological Process, Cellular Component and Molecular Function

GO ID	Description	***p***-value	Significant Associated Gene(s)
**Biological Process GO Terms**			
GO:0051153	regulation of striated muscle cell differentiation	0.0015	SOX6;MYOD1
GO:0051147	regulation of muscle cell differentiation	0.0030	SOX6;MYOD1
GO:0016202	regulation of striated muscle tissue development	0.0031	SOX6;MYOD1
GO:0048634	regulation of muscle organ development	0.0034	SOX6;MYOD1
GO:1901861	regulation of muscle tissue development	0.0034	SOX6;MYOD1
GO:1901700	response to oxygen-containing compound	0.0048	GPRC6A;RWDD1;KCNJ11;MYOD1
GO:0014070	response to organic cyclic compound	0.0071	RWDD1;KCNJ11;MYOD1
GO:0055026	negative regulation of cardiac muscle tissue development	0.0085	SOX6
GO:0048743	positive regulation of skeletal muscle fiber development	0.0085	MYOD1
GO:1905208	negative regulation of cardiocyte differentiation	0.0085	SOX6
**Cellular Component GO Terms**			
GO:0005887	integral component of plasma membrane	7.9726e-4	GPRC6A;CALHM4;CALHM5; CALHM6;KCNJ11
GO0031226	intrinsic component of plasma membrane	9.6402e-4	GPRC6A;CALHM4;CALHM5; CALHM6;KCNJ11
GO:0044459	plasma membrane part	0.0017	GPRC6A;CALHM4;CALHM5; CALHM6;FRK;KCNJ11
GO:0071944	cell periphery	0.0073	GPRC6A;CALHM4;CALHM5; CALHM6;COL10A1;FRK;KCNJ11
GO:0030008	TRAPP complex	0.0116	TRAPPC3L
GO:0030315	T-tubule	0.0132	KCNJ11
GO:0005886	plasma membrane	0.0276	GPRC6A;CALHM4;CALHM5; CALHM6;FRK;KCNJ11
GO:0005801	cis-Golgi network	0.0376	TRAPPC3L
GO:0022627	cytosolic small ribosomal subunit	0.0440	RPS13
GO:0005844	polysome	0.0488	RWDD1
**Molecular Function GO Terms**			
GO:0008146	sulfotransferase activity	0.0012	SOT3A1L;HS3ST5
GO:0016782	transferase activity, transferring sulfur-containing groups	0.0019	SOT3A1L;HS3ST5
GO:0008199	ferric iron binding	0.0091	FTL
GO:0034483	heparan sulfate sulfotransferase activity	0.0109	HS3ST5
GO:0070181	small ribosomal subunit rRNA binding	0.0127	RPS13
GO:0016722	oxidoreductase activity, oxidizing metal ions	0.0182	FTL
GO:0008198	ferrous iron binding	0.0182	FTL
GO:0030506	ankyrin binding	0.0182	KCNJ11
GO:0008253	5′-nucleotidase activity	0.0182	NT5DC1
GO:0005242	inward rectifier potassium channel activity	0.0182	KCNJ11

**Fig. 4:**
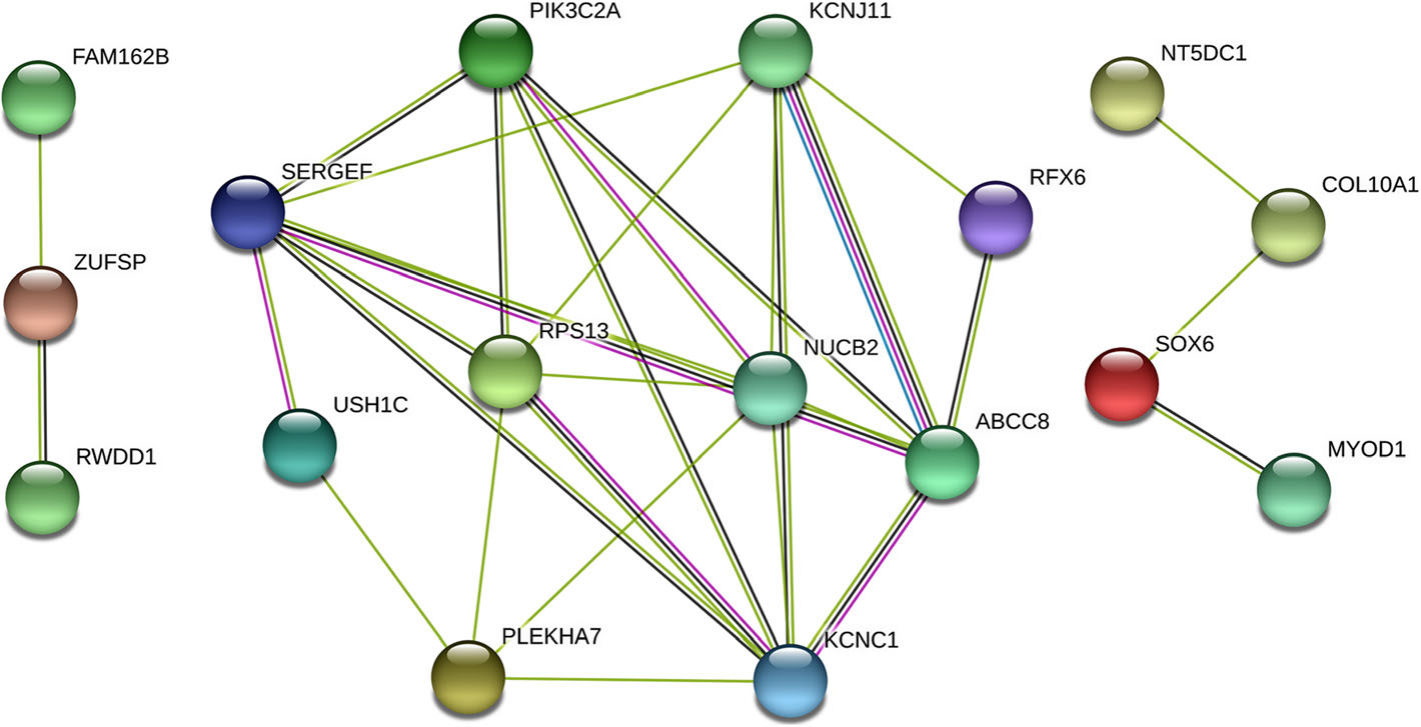
Confidence view of the network created by the STRING software. Nodes represent proteins produced by a single protein-coding gene locus. Edges represent protein-protein associations. Line colors indicate types of interaction evidence: known interactions from curated databases (cyan) or experimentally determined (pink); predicted interactions from gene neighborhood (green); and other sorts of interactions such as co-expression (black). The large network, in the middle, and smaller networks, on the right and left extremes, both relate to cell differentiation and muscle functioning

Complementary, STRING databases were used to search for enriched pathways and protein domains on genes annotated within 1-Mb window of significant CNV segments (Fig. 4 and Table 5). Interestingly, the three networks identified are related to cell differentiation and muscle functioning (Fig. 4).

In addition, terms associated with potassium channels and regulation of insulin secretion were enriched for CNV candidate genes related to performance traits (Table 5). Moreover, regarding to protein domains, the calcium homeostasis modulator family, consisting of three members of the *FAM26* gene family, was enriched. Furthermore, 78 publications significantly enriched in STRING are presented in Additional file 8.

**Table 5:** STRING enriched pathways and protein domains for CNV candidate genes related to performance traits

#term ID	Term Description	FDR^a^	Matching proteins IDs^b^	Matching proteins labels^c^
**Reactome Pathways**				
GGA-1296025	ATP sensitive Potassium channels	0.0043	ENSGALP00000009950, ENSGALP00000032081	ABCC8, KCNJ11
GGA-1296071	Potassium Channels	0.0145	ENSGALP00000009950, ENSGALP00000010023, ENSGALP00000032081	ABCC8, KCNC1, KCNJ11
GGA-1296065	Inwardly rectifying K+ channels	0.0214	ENSGALP00000009950, ENSGALP00000032081	ABCC8, KCNJ11
GGA-422356	Regulation of insulin secretion	0.0384	ENSGALP00000009950, ENSGALP00000032081	ABCC8, KCNJ11
**PFAM Protein Domains**				
PF14798	Calcium homeostasis modulator	3.35e-05	ENSGALP00000024076, ENSGALP00000024082, ENSGALP00000024083	FAM26D, FAM26E, FAM26F
**INTERPRO Protein Domains and Features**				
IPR029569	Calcium homeostasis modulator family	6.17e-05	ENSGALP00000024076, ENSGALP00000024082, ENSGALP00000024083	FAM26D, FAM26E, FAM26F

^a^False Discovery Rate

^b^matching proteins IDs in the network

^c^matching proteins labels in the network

## Discussion

To investigate the effect of CNVs on production-related traits in broilers, we analyzed a Brazilian broiler population, selected for body weight, carcass and cuts yield, feed conversion, fertility, chick viability and reduced abdominal fat. In addition, the availability of information about the family structure of this population allowed the identification of family-based CNVs.

CNVs are significant sources of genetic variation [39] and have been associated with disease, abnormal development, physical appearance as well as many other economic traits in livestock animals [6, 8, 31]. It is noteworthy that CNVs are generally in low LD with SNPs [40], and its taggability is lower than SNP taggability [41]. Therefore, the genetic variation explained by CNVs might not be fully captured in the traditional SNP-based analysis. Thus, CNV-based GWAS studies can provide valuable insights on the genetic control of economically important traits for livestock breeding programs. CNV mapping can be based on different reference genome assemblies, populations and platforms. Hence, variability of CNV breakpoints (i.e., genomic coordinates) can happen due to different biological and technical influences [11]. Therefore, CNV comparison among studies is not prosaic, even in the same species, and, as a consequence, different approaches may be complementary to each other [26, 42].

In our population, copy number gains were more abundant than losses. Likewise, Yi et al. [42], Gorla et al. [7] and Sohrabi et al. [43] reported more gains than losses and mixed regions in chicken populations. One reason is that duplications are more likely to be conserved than deletions because deletion regions are relatively gene-poor and therefore these regions are prone to purifying selection [44]. Nonetheless, deletion polymorphisms might have a significant role in the genetics of complex traits, even though not directly observed in several gene mapping studies [44].

In the present study, significant CNV segments associated with performance traits on chromosome 3, for body weight at 35, 41 and 42 days and body weight gain from 35 to 41 days, and on chromosome 5 for birth weight were identified. Given that these traits are not independent, and genetic correlations between performance traits have been widely reported in chickens [45], it is expected that certain CNV regions may be concomitantly associated with more than one trait, especially body weight measured in different ages (Fig. 1).

In the qPCR validation, we systematically assessed the overall agreement rate of the significant CNV segments detected in silico with qPCR results. The validation results indicated that all CNV segments were confirmed in at least one qPCR assay, consequently all CNVs may be real. Our results indicated that there is a small discrepancy (7.41%) between qPCR and PennCNV callings, which may be due to variations on the exact genomic coordinates of the CNVs that influenced the hybridization of the qPCR primers and the amplification efficiency.

We identified overlaps of significant CNV segments associated with body weight at 35, 41, 42 days and body weight gain with four previously mapped QTLs for weight traits and residual feed intake (RFI). RFI is defined as the difference between actual feed intake and predicted feed intake based on energy requirements for body weight gain and maintenance [46]. Moreover, we found genomic windows defined by significant CNV segments overlapping published QTLs for growth-related traits in the Embrapa F2 Chicken Resource Population [24]. Many studies, conducted with different chicken lines, have successfully identified QTLs and genes associated with economically important traits [47]. Given that QTLs and genes underlie functional regions of the genome, they may not be prone to structural rearrangements and thus not expected to be subject to CNVs [23]. Therefore, QTLs and genes located inside or nearby CNVs are of special interest.

Noticeably, SNP-based studies [37, 46, 48] have identified many more QTLs associated with the traits analyzed in our study than the CNV-based approach applied here. Indeed, Pértille et al. [37] identified 88 QTLs associated with feed conversion, feed intake, birth weight, and body weight at 35 and 41 days of age in the Embrapa F2 Chicken Resource Population. Mebratie et al. [46] and Moreira et al. [48] identified, respectively, 11 and 19 QTLs associated with body weight traits in a commercial broiler chicken population and in the Embrapa F2 Chicken Resource Population. This difference in QTL mapping is expected since CNVs are more frequently associated with deleterious effects than favorable ones, which is not the case of SNPs, at least those included in the SNP arrays [49]. In addition, since known QTLs were (mostly) mapped using microsatellite markers and SNPs, they will not necessarily capture the same effect as CNVs. If associated CNVs do not overlap with QTLs previously found in other studies, that could occur because specific CNV probes can be excluded from a SNP-GWAS due to Hardy-Weinberg equilibrium deviation or rigorous multiple testing corrections [23].

CNVs that comprise functional genes may induce phenotypic variation by altering gene structure, dosage and regulation, as a consequence of natural evolutionary processes [50], such as genetic drift or artificial selection. We identified 32 genes annotated within a 1-Mb window of significant CNV segments associated with birth weight, body weight at 35, 41 and 42 days and body weight gain from 35 to 41 days.

Note that animals presenting deletions (0n/1n) in significant CNV segments were less frequent in our population, while their average body weights at birth and at 35, 41 and 42 days of age were higher compared to animals with normal copy number (2n) in the same CNV segments (Fig. 2). Two reasons may explain the low frequency of favorable genotypes for body weights at different ages: 1) this meat-type population has been under multiple trait selection, not exclusively focused on improving body weight, and 2) the TT line that gave rise to the TT Reference Population was selected for only 17 generations [51]. In 2010, Johansson et al. [52] conducted a study with two chicken lines (high and low body weight lines) from a single trait selection experiment, where even after 50 generations of selection, the high line is still responding to selection. Conversely, for body weight gain, the increase in the copy number of the respective significant CNV segment was positively associated with the phenotype (Fig. 2). Since these CNV segments are located in proximity of several genes (Table 3) and, as it has been shown that the expression of a gene may be affected by their presence [12], CNVs may act as important modulators of gene expression.

CNVs inserted in regulatory regions like enhancers, promoters or in 3’UTR regions, may modify availability of binding sites to transcription factors or miRNAs, respectively, resulting in the modulation of their associated genes. In addition, a wide variety of cis-regulatory elements have been investigated for the presence of CpG islands and methylation. Despite being frequently found in promoter regions and TSSs, low levels of methylation can be found in enhancers and insulators, modulating gene transcription [53]. However, as expected due to its low frequency distribution in the genome, no overlap between CpG islands and the significant CNV segments was identified.

Our analysis of ChIP-seq data suggests that the significant CNV segment located on chromosome 5 may be affecting expression of its cognate genes due to the presence of regulatory elements (Additional file 6). Once enhancer activity is tissue-specific, our analysis does not reject the possibility that these elements may be present in other tissues related to metabolism and weight gain, like hepatic and adipose tissues [54, 55]. In addition, although the other significant segments have not shown peaks for enhancers, it does not mean that other regulatory elements, such as insulators and silencers, cannot be associated and affect gene expression. In this sense, CNVs may have potential to modulate core regulators located in their proximity, propagating such effects to genome-wide gene expression [12] and accounting for differences manifested at the phenotype level (Fig. 2). Near to the CNV segment on chromosome 5, we identified *KCNJ11*, *MyoD1*, *PIK3C2A* and *SOX6* genes, which might have important effects on chicken growth and development regulation.

The *KCNJ11* gene, found to be 2217 base pairs upstream of the aforementioned significant CNV segment, is known to regulate insulin secretion [56]. A glucose metabolism disorder is usually linked as a cause of reduced development of chicken muscle tissue under stress, especially in broilers [57]. This gene was enriched for the ATP-sensitive potassium channel (KATP) pathway. KATP subunits are, among other genes, encoded by *KCNJ11*. KATP channels have a high activity in rat fast-twitch fibers, distinguished by raised muscle strength, and a low activity in slow-twitch fibers, characterized by weakness, fragility and lowered muscle strength [58]. *KCNJ11* gene knockout mice present reduced glycogen, slender phenotype and weakness [58]. It has been reported that the effect of the *KCNJ11* gene on muscle may occur due to alterations in the KATP channel activity, which, in turn, affects the potassium flow inside the cell, settling the membrane potential needed for muscle activity [59]. For this reason, this gene may promote early growth and development in chickens. In fact, it was found to be highly expressed in the muscle tissue of one-week-old chicks [56], which supports our findings that this gene, closely located to a significantly associated CNV segment, can play a role in the regulation of birth weight. Our analysis of regulatory elements suggests that changes in the copy number in the *KCNJ11* gene might be affecting gene transcription and could explain the effect on birth weight associated with these changes. This hypothesis, however, needs to be addressed by RNA data on different tissues. Interestingly, a novel 163-bp indel in the downstream region of this gene was significantly associated with growth traits in chickens [56]. In the same study, synteny analyses found that *KCNJ11* maintains a close connection with its neighboring genes. It is interesting to note that one of these genes is the Myogenic differentiation 1 (*MyoD1*).

The myogenic regulatory factors are a family of vertebrate proteins (*MyoD*, *Myf5*, *Mrf4* and *Myog*) that are robust transcription factors for muscle genes [60]. The *MyoD1* gene can promote myoblast differentiation and have relevant effects on muscle development [61]. Previous study in quail lines revealed that a delay in *MyoD1* expression is associated with increased body weight and muscle mass [62]. A very high degree of synteny is maintained between *MyoD1* containing regions of human chromosome 11 and chicken chromosome 5, comprising *ABCC8*, *KCNJ11*, *PIK3C2A*, *RPS13*, *SERGEF*, *NUCB2* and *PLEKHA7* genes [63]. Mutations in *PIK3C2A* gene were discovered to cause a growth-related genetic syndrome in humans, consisting of dysmorphic features, short stature and skeletal abnormalities [64]. This gene has been attributed to biological functions such as glucose transport, Akt pathway activation, endosomal trafficking, phagosome maturation, mitotic spindle organization, exocytosis and autophagy [65].

*MyoD1* was enriched in biological processes associated with the *SOX6*, a gene related with muscle physiology, such as regulation of striated muscle cell differentiation and development, regulation of muscle cell differentiation and regulation of muscle organ development. The expression level of the *SOX6* gene was positively associated with CNV and increased during skeletal muscle cell differentiation, by upregulating expression levels of muscle-growth-related genes in chickens as well as in other animal species [66].

We found genes nearby significant CNV segments associated with body weight at 35, 41 and 42 days (*LPIN1* and *TRIB2*) and body weight gain (*GPRC6A* and *NT5DC1*) that may be of special importance and have potential effects on chicken growth. A significant association was found between a variant in the 3′ UTR of chicken *LPIN1* gene and breast muscle fiber diameter [67], suggesting that this gene has a potential effect on muscle fiber development. The *TRIB2* gene, a novel regulator of thymocyte cellular proliferation, was found to be involved in reproduction and growth in White Leghorn chickens, and consequently might represent footprints of the selection process [68]. The *GPRC6A* gene was found to have functions related to testis growth and development in broilers [69]. In addition, another interesting gene is the *NT5DC1*, previously related to muscle tissue, angiogenesis and amino acid metabolism [70]. We found an enriched cluster for calcium homeostasis modulator (*CALHM*) gene family, which included three members: *FAM26D*, *FAM26E* and *FAM26F*. Even though *CALHM*s have been classified as pore-forming subunits of plasma membrane ion channels, questions about their function remain unanswered, hence their role needs to be ascertained on further investigations [71].

In summary, from SNP-chip data of a broiler population, we identified novel structural variation regions in the genome that, based on gene enrichment and literature information, harbor potential candidate genes, with important roles in a wide range of biological, cellular, and molecular processes, linked with muscle differentiation, growth, and development. Our findings reveal that alterations in copy number within or nearby these genes could result in phenotypic variation, thus contributing to a better understanding of performance regulation in chickens.

## Conclusions

This study identified structural variations associated with five complex traits of interest in a broiler population using a probe-level based CNV association approach. Our results provide substantial information about the potential CNV impacts on animal production, growth, development, and performance-related traits, laying a foundation for incorporating CNVs into the future poultry breeding programs and contributing to expand scientific research on genetics, particularly on structural variations involved in animal biology and physiology.

## Methods

### Population description

A paternal broiler line (TT) belonging to the Chicken Breeding Program of Embrapa Swine and Poultry National Research Center, in Concórdia, Santa Catarina State, South of Brazil, was developed in 1992. This line originated from White Plymouth Rock and White Cornish breeds and has been under multiple trait selection to improve body weight, feed conversion, carcass and breast yields, viability, fertility, reduction of abdominal fat and metabolic syndromes [51]. The experimental broiler population evaluated in this study, called TT Reference Population, was generated by an expansion of the paternal broiler line TT and consisted of approximately 1500 chickens, which were all slaughtered in 2008, generated in five hatches from 20 males and 92 females (1:5). More details can be found in Marchesi et al. [51].

### Phenotype measurement

Body weight was recorded at 1 (birth weight), 21, 35, 41 and 42 (after fasting) days of age. Over the period between 35 and 41 days of age, chickens were transferred to individual cages for measuring feed intake and body weight gain, to evaluate feed conversion. At 42 days of age, all chickens (~ 1500 individuals) were weighted and euthanized by cervical dislocation followed by exsanguination. By then, a blood sample from each animal was collected for subsequent DNA extraction. In this study, we analyzed eight performance traits: (i) birth weight (BW), (ii) body weight at 21 days of age (BW21), (iii) body weight at 35 days of age (BW35), (iv) body weight at 41 days of age (BW41), (v) body weight at 42 days of age (BW42), (vi) feed intake measured from 35 to 41 days of age (FI), (vii) feed conversion ratio measured from 35 to 41 days of age (FCR) and, (viii) body weight gain measured from 35 to 41 days of age (BWG). More detailed descriptions on this population, rearing conditions and phenotype measurements are available in Marchesi et al. [51]. The descriptive statistics for the analyzed phenotypes are shown in Table 6.

**Table 6:** Descriptive statistics from phenotypic values for performance traits analyzed in the TT Reference Population

Traits^a^	N^b^	Mean	SD^c^	Minimum	Maximum
BW	1448	47.66	3.70	37.40	61.80
BW21	1426	648.43	133.86	256	1034
BW35	1450	1730.96	202.52	776	2444
BW41	1443	2219.20	251.82	1026	2922
BW42	1452	2223.86	260.15	988	2971
FI	1443	1091.45	152.43	508	1590
FCR	1439	2.31	0.47	1.42	5.25
BWG	1439	488.77	106.53	128	802

^a^BW: birth weight in grams; BW21: body weight at 21 days in grams; BW35: body weight at 35 days in grams; BW41: body weight at 41 days in grams; BW42: body weight at 42 days in grams; FI: feed intake from 35 to 41 days in grams; FCR: feed conversion ratio from 35 to 41 days; BWG: body weight gain from 35 to 41 days in grams

^b^Number of animals

^c^Standard deviation of the mean

### DNA extraction, genotyping and quality control

Genomic DNA from 1461 blood samples was extracted using the PureLink® Genomic DNA (Invitrogen, Carlsbad, CA, USA) kit and then quantified using Qubit® 2.0 Fluorometer (Thermo Fisher Scientific, Waltham, MA, USA). After extraction, DNA integrity was evaluated on agarose gel (1%) and diluted to 10 ng/μL. Diluted genomic DNA was prepared following recommended Affymetrix protocols in order to perform the genotyping analysis using the 600 K Affymetrix Axiom Genotyping Array (Affymetrix®, Inc. Santa Clara, CA, USA, [49]), that contains segregating SNPs for different populations, including commercial broiler lines.

Initially, Axiom™ Analysis Power Tools (Affymetrix®) software v.2.10.2.2 was used to filter genotypes based on DishQC and call rate parameters. A minimum default quality control of 0.82 and a minimum sample call rate of 97% were used. Therefore, only samples with DishQC ≥0.82 and call rate ≥ 97% were kept for following analyses. SNPs in sex chromosomes, and those not mapped in the chicken genome assembly (GRCg6a) were excluded. Only SNPs annotated to autosomal chromosomes from GGA1 to GGA33 were included in the analysis.

From a total of 1461 genotyped chickens, 223 samples were removed from the analysis after applying the DishQC criteria, and a filter on sample call rate ≥ 97% loci. From the total of 580,961 SNPs available on the SNP array, 476,254 informative polymorphic SNPs on the autosomal chromosomes (GGA1–33) were kept after filtering.

### Input construction and CNV calling

CNV calling was performed using PennCNV v.1.0.5 [20], an integrated hidden Markov model (HMM) that merges various sources of information, including relative signal intensities (log R Ratio, LRR) and relative allele frequencies (B allele frequency, BAF) at each SNP, the distance between adjacent SNPs, and the population frequency of the B allele (PFB).

The files denominated ‘summary’, ‘calls’ and ‘confidences’ that are built during SNP genotyping and initial data filtering, and are required for signal intensities estimation, were used to extract the LRR and BAF values. First, these files were used to generate canonical clusters [72] by the PennCNV-Affy ‘generate_affy_ geno_cluster.pl’ function, which allows the estimation of the LRR and BAF values by the PennCNV-Affy ‘normalize_affy_geno_cluster.pl’ function. Then, the PFB file was estimated from marker’s individual BAF values, using the PennCNV ‘compile_pfb.pl’ function. Next, the individual-based CNV calling was performed using the -test option with default parameters for the HMM model. Given that the GC ratio content around each SNP marker is known to influence signal strength, creating the so-called genomic waves [73], the LRR of each sample was corrected using the chicken GC content file (i.e., GC content of 1-Mb genomic regions surrounding each SNP) by the -gcmodel option. As long as family structure can be used for generating more accurate CNV calls [20], and pedigree information for a father-mother-offspring trio was available, a family-based CNV detection algorithm was used to jointly update CNV status previously obtained in the individual-based calling step.

For CNV filtering, the default PennCNV standard deviation (SD) criteria for LRR ≤ 0.35, BAF drift< 0.01, and waviness factor ≤ 0.05 were used. Note that the waviness factor represents the dispersion in signal intensity over the genome. Following, CNVs with minor allele frequencies (MAF) less than 0.05 were removed to avoid calling artefacts. Moreover, CNVs smaller than 1 kb were also excluded and only CNVs consisting of at least three consecutive SNPs were retained in the analysis [7]. Lastly, all duplicated CNVs (i.e., same event in the same parental) were removed. Duplicated CNV entries occurred due to half sib families, as some sires and dams were included more than once in PennCNV analysis. The CNV calling was focused only on autosomal chromosomes GGA1 to GGA33 as PennCNV results for sex chromosomes are unreliable and difficult to interpret [20].

### CNVR compilation

Individual CNV calls filtered by PennCNV overlapping at least one base pair were concatenated into CNV regions (CNVRs) using the populationRanges (grl, density = 0.1) function from the CNVRanger R/Bioconductor package [74]. Genomic areas with density < 10% were deleted to avoid false positive predictions. The CNVRs were classified as gain or loss. The overlapping CNVRs of ‘gain’ and ‘loss’ were merged into single regions to account for genomic regions in which both events can occur (i.e., ‘both’ CNVRs). The frequency of each CNVR was estimated based on the number of samples mapped at the genomic interval covered by the CNVR.

### Genome-wide association analyses

Genome-wide association analyses between performance traits and CNV segments were carried out using the CNVRanger R/Bioconductor package [74]. This procedure was originally proposed by da Silva et al. [23] . First, the CNV segments to be used in the association analyses were established. For that, a state was assigned for each of the SNP probes overlapping a CNV call. Then, we estimated the CNV frequency in each probe and selected only those with frequency above 5% [74]. Finally, selected probes were used to construct the CNV segments based on a CNV-genotype similarity, in which subsequent probes with identical genotype in ≥95% of our population were concatenated to CNV segments. A raw *p*-value was independently generated for each probe. Following, the raw *p*-values were corrected using genomic inflation and, the probe with the lowest *p-*value was selected to represent the CNV segment. Multiple testing correction was carried out using the FDR method [75] generating the *q*-values for each CNV segment. The following statistical models were used for association analyses:

- *y*_*ijkl*_ = *μ* + *S*_*i*_ + *H*_*j*_ + *CNV*_*k*_ + *a*_*l*_ + *e*_*ijkl*_
- $$ {y}_{ijklm}=\mu +{S}_i+{H}_j+{CNV}_k+b\left( BW{35}_l-\overline{BW35}\right)+{a}_m+{e}_{ijklm} $$

where *y*_*ijkl*_ and *y*_*ijklm*_ are the phenotypic record on the *l*^th^ or *m*^th^ animal, respectively, *μ* is the overall intercept, *S*_*i*_ is the fixed effect of the *i*^th^ sex (i = 1, 2), *H*_*j*_ is the fixed effect of the *j*^th^ hatch (j = 1, 2, 3, 4, 5), *CNV*_*k*_ is the number of copies of a given allele in the genotype of the *k*^th^ CNV segment (k = 1, …, 191, represented as gain, loss and normal (2n), and coded as 1, − 1 and 0, respectively), *b* is the linear regression coefficient related to the BW35 effect considered as deviation from the mean ($\overline{BW35}$), *a*_*l*_ and *a*_*m*_ are the random direct additive genetic effect for the *l*^th^ or *m*^th^ animal, respectively, and *e*_*ijkl*_ and *e*_*ijklm*_ are the random residual effect for the *l*^th^ or *m*^th^ animal, respectively. Note that sex and hatch were included in the models as class effects for all phenotypes, and BW35 was fit as continuous effect only for FI, FCR and BWG (model b). The random components of the models were distributed as $\mathbf{a}\sim \mathrm{N}\left(0,\mathbf{G}{\upsigma}_{\mathrm{a}}^2\right)$ and $\mathbf{e}\sim \mathrm{N}\left(0,\mathbf{I}{\upsigma}_{\mathrm{e}}^2\right)$, where ${\upsigma}_{\mathrm{a}}^2$ and ${\upsigma}_{\mathrm{e}}^2$ are the genetic and residual variances, respectively, **G** is the CNV-based genomic relationship matrix, and **I** is an identity matrix.

Lastly, we established two different thresholds. The first corresponded to a suggestive association (FDR-corrected *p*-value< 0.1) and should be used to identify CNVs for consideration in future studies. The second one corresponded to a significant association (FDR-corrected *p*-value< 0.05), consequently, highlighting regions more likely to be truly associated with the investigated phenotypes [76].

### Validation by qPCR

Quantitative PCR (qPCR) was carried out to validate the significant CNV segments associated with performance traits. Copy number was determined in the 3 significant CNV segments using primer pairs designed to target each segment. It is noteworthy that 3 different primer pairs were designed for each CNV segment to account for possible breakpoints variations. A total of 18 samples, consisting of 3 reference animals (2n) and 3 different testing animals per CNV segment, were selected for the validation process based on the amount of double-stranded DNA (dsDNA) measured with Qubit® 2.0 Fluorometer. Primers designed using Primer3plus [77] were quality tested through NetPrimer (http://www.premierbiosoft.com/netprimer). Additionally, we used the SNPdb [78] against the Ensembl-Biomart tool (http://www.ensembl.org/biomart/martview, [79]) to check the presence of SNPs in the genomic region targeted by the primers.

All primers were previously PCR-tested to verify non-specific amplicons and to optimize qPCR conditions. A qPCR solution of a final 10 μl was used consisting of 5.0 μl PowerUp™ SYBR™ Green Master Mix 2x (Applied Biosystems®, catalog number: A25742), 0.5 μl forward primer (10 mM), 0.5 μl reverse primer (10 mM) and 4.0 μl of genomic DNA (2.5 ng/μl). The reference and testing samples were amplified with the designed primers sets in technical triplicates carried out in QuantStudio™ 12 k Flex machine coupled to QuantStudio 12 K Flex Software v.1.2.2 (Applied Biosystems®). The qPCR thermocycling steps were as follows: 50 °C for 2 min, 95 °C for 2 min and 40 cycles of amplification (95 °C for 15 s, 55–60 °C (primer-dependent) for 15 s and 72 °C for 1 min). The reference samples were randomly chosen from a set predicted by PennCNV to have normal copy number status on all the tested regions.

Cycle thresholds (Ct) were corrected by primer mean efficiency calculated by LinReg [80] and copy number was estimated from normalized ratio method (NR): 2 × 2^-(∆∆Ct)^ [81]. The primers for the propionylcoenzyme A carboxylase gene (PCCA, GGA1) were used as references [82]. Moreover, the control value was estimated based on the average value of ΔCt from reference diploid animals, and copy number states were categorized based on the geometric mean between copy number 1, 2 and 3 [83], where lack of amplification was considered as 0n (complete deletion).

### CNV segments overlapping known QTLs

Overlaps of the significant associated CNV segments with previously mapped QTLs for performance traits were determined using information from the Chicken QTLdb - release 43 (https://www.animalgenome.org/cgi-bin/QTLdb/GG/index, [5]. We used the available .bed files with the QTL coordinates based on the GRCg6a genome assembly to check for overlaps using the subsetByOverlaps function from the GenomicRanges R/Bioconductor package [84]. All previously mapped QTLs were reported by QTL ID numbers, available at the Chicken QTLdb [5].

Additionally, we checked the overlapping between the genomic windows covered by the significant CNV segments and the QTLs for growth-related traits reported for the Embrapa F2 Chicken Resource Population, which was originated by crossing sires of the paternal broiler line TT (same line used to obtain the TT Reference Population) and dams of a maternal layer line [37]. The genomic coordinates of the reported QTLs were converted from Gallus_gallus-5.0 to GRCg6a using the LiftOver tool (https://genome.ucsc.edu/cgi-bin/hgLiftOver).

### CNV segments overlapping regulatory elements

The overlaps of the significant associated CNV segments with CpG islands were determined using information from the GRCg6a genome assembly, available on UCSC Genome Browser (http://hgdownload.soe.ucsc.edu/goldenPath/galGal6/bigZips/). In addition, NCBI Refseq data, equally obtained from the UCSC Genome Browser (http://hgdownload.soe.ucsc.edu/goldenPath/galGal6/bigZips/genes/galGal6.ncbiRefSeq.gtf.gz), were used to locate TSSs (Transcription Start Sites) in the significant CNV segments and infer promoter regions based on their genomic location (2 kb upstream of a TSS).

Moreover, to check for overlaps of the significant segments with enhancers, we used publicly available H3K27ac ChIP-seq (Chromatin Immunoprecipitation and sequencing) data from two replicates of chicken skeletal muscle, obtained from the Functional Annotation of Animal Genomes Consortium (FAANG, https://www.ncbi.nlm.nih.gov/geo, GSM4799754 and GSM4799755). First, reads were trimmed with Trim Galore! 0.6.5 (https://github.com/FelixKrueger/TrimGalore) using the default arguments, and then aligned with BWA mem 0.7.17 [85] to the GRCg6a genome. The alignments were sorted and indexed with Samtools 1.11 [86] using the arguments -F 1804 and -q 30. We marked the duplicates with Picard toolkit 2.25.1 (http://broadinstitute.github.io/picard/) and removed them with Samtools. Then, aligned reads in .bam files were converted to .bedgraph. The .bed files with significant peaks (−q 0.01) were obtained from GEO (https://www.ncbi.nlm.nih.gov/geo, GSE158430) and then, the final data (.bedgraph and .bed files) were uploaded in IGV (Integrative Genome Viewer) [87] for visualization.

### Identification of candidate genes and gene-set analysis

The gene content of significant CNV segments was assessed using Ensembl Release 101 BioMart tool (https://www.ensembl.org/biomart/martview, [79]), based on the GRCg6a genome assembly. We investigated genes flanking genomic intervals for the significant associated CNV segments, corresponding to 1 Mb windows (500 kb up and downstream).

Enrichment analyses were performed with WebGestalt (http://www.webgestalt.org/), a “WEB-based GEne SeT AnaLysis Toolkit” designed for functional genomics, proteomics and large-scale genetic studies [88]. GO-terms for biological process (BP), cellular component (CC) and molecular function (MF) were investigated. Multiple testing correction was carried out using the default option (i.e., Benjamini-Hochberg method [75]). In addition, STRING v.11 (http://string-db.org/, [89]) was used as a complementary approach to search for enriched pathways and protein domains. Conversely to WebGestalt, the STRING database intends to integrate all publicly available sources of protein–protein direct and indirect interaction information to obtain a comprehensive global network.

## Supplementary Information

**Additional file 1.** Information of identified CNVRs

**Additional file 2 **Manhattan plots for CNV segments across the 33 autosomal chromosomes associated with (a) birth weight, (b) body weight at 35 days, (c) body weight at 41 days and (d) body weight at 42 days and (e) body weight gain. The X-axis represents the somatic chromosomes, and Y-axis shows the corresponding -log10 *p*-value. Red and blue lines indicate *p*-values of 0.05 and 0.1, respectively

**Additional file 3 **QQ-plots show the relation of normal theoretical quantiles of the probability distributions between expected (X-axis) and observed (Y-axis) *p*-values from (a) birth weight, (b) body weight at 35 days, (c) body weight at 41 days, (d) body weight at 42 days and (e) body weight gain

**Additional file 4.** Information of the CNV segments validated by qPCR and the primers used for qPCR

**Additional file 5.** Validation rates of 9 samples

**Additional file 6.** H3K27ac and CpG islands tracks alingment with significant CNV segments. H3K27ac ChIP-seq data of chicken skeletal muscle (blue and red tracks) overlap with the significant CNV segment on chr5:12059966–12062666 (green bar) near the KCNJ11 gene (black bar). The blue rows represent replicate A, while red rows represent replicate B. The overlap with the H3K27ac enriched region is indicated by the dashed box. No overlap was found between the significant CNV segments on chr3:64169030–64171297 or chr3:97801202–97809208 and H3K27ac regions. CpG islands were only located in promoter regions and TSSs (orange track)

**Additional file 7.** List with the 32 genes annotated within a 1-Mb window of significant CNV segments

**Additional file 8.** 78 publications significantly enriched in the STRING network

## Data Availability

All data generated and/or analyzed during this study are public and included in this published article. The chicken genome assembly (GRCg6a) was retrieved from the UCSC Genome Browser website (http://hgdownload.soe.ucsc.edu/goldenPath/galGal6/bigZips/). All previously mapped QTLs were reported by QTL ID numbers, available at the Chicken QTLdb (https://www.animalgenome.org/cgi-bin/QTLdb/GG/index). NCBI Refseq data was obtained from the UCSC Genome Browser (http://hgdownload.soe.ucsc.edu/goldenPath/galGal6/bigZips/genes/galGal6.ncbiRefSeq.gtf.gz). The H3K27ac ChIP-seq data are publicly available at the Functional Annotation of Animal Genomes Consortium repository (FAANG, https://www.ncbi.nlm.nih.gov/geo, GEO accession GSM4799754 and GSM4799755). The datasets used and/or analyzed during the current study (genotypes and phenotypes) are available from the corresponding author on reasonable request.

## Abbreviations

aCGH: Comparative Genomic Hybridization array
BAF: B Alelle Frequency
BW: Birth Weight
BW21: Body Weight at 21 days of age
BW35: Body Weight at 35 days of age
BW41: Body Weight at 41 days of age
BW42: Body Weight at 42 days of age
BWA: Burrows-Wheeler Aligner
BWG: Body Weight Gain
ChIP-seq: Chromatin Immunoprecipitation and sequencing
CNV: Copy Number Variation
CNVR: Copy Number Variation Region
Ct: Cycle threshold
dsDNA: double-stranded DNA
FAANG: Functional Annotation of Animal Genomes
FCR: Feed Conversion Ratio
FDR: False Discovery Rate
FI: Feed Intake
GO: Gene Ontology
GWAS: Genome-Wide Association Study
H3K27ac: Histone H3 acetylated at lysine 27
IGV: Integrative Genome Viewer
KATP: ATP-sensitive potassium channel
LD: Linkage Disequilibrium
LOH: Loss of Heterozygosity
LRR: Log R Ratio
MAF: Minor Allele Frequency
NCBI: National Center for Biotechnology Information
PCCA: propionylcoenzyme A carboxylase
PFB: Population Frequency of the B Allele
qPCR: quantitative Polymerase Chain Reaction
QTL: Quantitative Trait Locus
RFI: Residual Feed Intake
SNP: Single Nucleotide Polymorphism
STRING: Search Tool for the Retrieval of Interacting Genes/Proteins
TSS: Transcription Start Site
USCS: University of California Santa Cruz
WebGestalt: WEB-based GEne SeT AnaLysis Toolkit
